# Free Tissue Transfer Versus Locoregional Flaps for the Reconstruction of Small and Moderate Defects in the Head and Neck Region: A Narrative Review

**DOI:** 10.7759/cureus.84711

**Published:** 2025-05-23

**Authors:** Alexandros Louizakis, Asterios Antoniou, Ioanna Kalaitsidou, Dimitris Tatsis

**Affiliations:** 1 Department of Oral and Maxillofacial Surgery, Aristotle University of Thessaloniki, Specialized Cancer Treatment and Reconstruction Centre, General Hospital of Thessaloniki "George Papanikolaou", Thessaloniki, GRC; 2 Department of Cranio-Maxillofacial Surgery, Inselspital, Bern University Hospital, University of Bern, Bern, CHE

**Keywords:** functional outcomes, head and neck reconstruction, locoregional flaps, microvascular free flaps, oral cavity defects

## Abstract

Reconstructive surgery for oral cavity defects has progressed from early pedicled locoregional flaps, like the pectoralis major myocutaneous flap, to sophisticated microvascular free flaps, driven by the need to restore critical functions such as speech, swallowing, and chewing, alongside aesthetic outcomes essential for patient quality of life. This narrative review compares the effectiveness, outcomes, and current roles of microvascular free flaps versus locoregional flaps in reconstructing small to moderate oral cavity defects. A narrative literature review was conducted, analyzing retrospective studies, meta-analyses, and clinical series, focusing on flap success rates, functional and aesthetic outcomes, complications, and resource utilization for key flaps, including radial forearm free flap (RFFF), anterolateral thigh flap (ALT), submental island flap (SMIF), supraclavicular artery island flap (SCAIF), and facial artery musculomucosal (FAMM) flap. Microvascular free flaps achieve high success rates and excel in complex three-dimensional reconstructions, offering superior functional outcomes, but demand prolonged operative times, specialized expertise, and significant resources, limiting their feasibility in low-resource settings. Locoregional flaps provide comparable success for smaller defects, with shorter operative times, lower costs, and suitability for high-risk patients. Both approaches yield favorable aesthetic results when appropriately selected, with locoregional flaps offering better tissue matching in facial reconstructions. Free flaps remain the gold standard for complex defects, while locoregional flaps are effective, cost-efficient alternatives for smaller defects, particularly in comorbid patients or resource-constrained environments. Clinical decisions should consider defect complexity, patient health, and institutional capabilities, with future advancements in tissue engineering and surgical training poised to enhance outcomes and accessibility. The aim of this review is to clarify the differences between the traditionally used locoregional flaps and the more recent microvascular free flaps.

## Introduction and background

Background on oral cavity defects

Tracing back the history of head and neck reconstruction, various locoregional flaps have been successfully used, both pedicled flaps (axial) and random pattern flaps [1]. Chronologically, the first pedicled flap ever used in head and neck surgery was a forehead flap, followed by the pectoralis major myocutaneous flap (PMMF), which is supplied by a branch of the thoracoacromial artery and was considered the gold standard for decades. Later, other flaps such as the submental island flap (SMIF) and the supraclavicular island flap (SCIF) found their role in head and neck surgery [2,3]. Other popular locoregional flaps that are still widely being used in reconstructing small to large defects are the submental island artery flap and the facial artery musculomucosal (FAMM) flap [4,5]. Moreover, the platysma myocutaneous flap and the sternocleidomastoid muscle can be very useful in plenty of cases [6,7].

On the other hand, the gold standard nowadays is the use of microvascular free flaps [8]. There are various types of free flaps that can be used depending on the site of resection and the defect that needs to be replaced. Those can be fasciocutaneous, osteocutaneous, or myocutaneous [9]. The most common free flaps used are the radial forearm free flap (RFFF), which is the workhorse in small and medium oral defects, and also the anterolateral thigh flap (ALT) and the latissimus dorsi in larger defects [8,10]. When bone reconstruction is of utmost importance, the fibula flap, the scapular and parascapular flaps, and the deep circumflex iliac artery (DCIA) flap can also be used [11]. Chimeric flaps, which refer to flaps composed of multiple tissue components (e.g., skin, muscle, and bone) that are harvested on separate branches of the same vascular pedicle, allow for independent positioning of each component while maintaining a single blood supply and can be utilized in larger and more complex defects in the head and neck region [8,10]. As a result, better functional and cosmetic outcomes can be achieved by the use of these advanced flaps [12].

Importance of reconstruction: emphasize the functional and aesthetic importance of effective reconstruction

Effective reconstruction of oral cavity and perioral defects is critical for both functional and aesthetic outcomes, significantly impacting patients' quality of life. Functionally, reconstruction restores essential capabilities such as speech, swallowing, and chewing, which are often compromised following surgical resection of tumors or trauma [13]. Aesthetically, reconstruction aims to preserve or restore facial symmetry and appearance, which are vital for psychological well-being and social interactions [14]. Techniques like microvascular free flaps allow for precise tissue matching and contouring, minimizing visible scarring and deformities. By addressing both functional and aesthetic needs, effective reconstruction enhances patient confidence and facilitates an improved quality of life [15].

Evolution of reconstructive techniques: a brief historical perspective on the development of free and locoregional flaps

The development of reconstructive techniques for oral cavity defects has evolved significantly, transitioning from basic locoregional flaps to sophisticated microvascular free flaps [16]. Historically, the earliest recorded use of a pedicled flap was the forehead flap, followed by the PMMF in the 20th century, which became a cornerstone due to its reliability and robust blood supply [17]. Other locoregional flaps, such as the SMIF and FAMM flap, emerged to address smaller defects with improved aesthetic outcomes [18]. The advent of microvascular free flaps in the late 20th century marked a paradigm shift, offering greater versatility and precision. Flaps like the RFFF and ALT became preferred for their adaptability to various defect sizes, while osteocutaneous flaps like the fibula flap revolutionized bone reconstruction [19,20].

Objective of the review

Various types of flaps have been used over the years in head and neck reconstruction after the ablation of tumors in this region. The aim of this review is to clarify the differences between the traditionally used locoregional flaps and the more recent microvascular free flaps.

## Review

Overview of the flap types

Free Flaps

Great progress has been achieved over the past few years in the field of microsurgery. Initially, free flaps were not considered the first option regarding the reconstruction of the head and neck area. They were mostly used in special cases of extended deficits, especially where the other known available options such as the locoregional flaps or the free tissue transfer failed to achieve the desired results both in head and neck oncology and in acute trauma [21,22]. However, advances in microsurgery led to the great use of microvascular free flaps in the past few years, making this option much more popular and easier to use [23].

Microsurgery encircles all the surgical procedures performed under a microscope. It refers to the surgical anastomosis of not only microvascular but also lymphatic or microneural structures. The diameter of these structures is usually less than 3 mm [21]. The advances in the latest years in the field of surgical equipment, moving from operating loops to high-resolution microscopes and specialized microsurgical instruments, as well as the wider use of anticoagulants, made these operating methods much more approachable and desirable by the wide surgical community [10,21,22].

On the other hand, the dexterity and the extensive practice in microsurgery that is needed by the surgeon, regarding the simulation in vein and artery grafting, as well as the high cost of the surgical equipment, still limit the practice of microsurgery in specialized reconstructive centers [21].

Locoregional Flaps

As already mentioned, in the last few years, free flaps have been proposed as the gold standard for this purpose [4]. However, not all cases of head and neck cancer can be treated with microvascular free flaps. Free flap reconstruction often involves demanding surgeries, requiring large operating times, highly trained personnel, and also expensive microvascular [4]. Moreover, not all patients are good candidates for free flap reconstruction. Not only the greater operation time and the prolonged patient recovery but also the need for intensive care unit (ICU) admittance make those flaps a less desirable method for patients with concomitant diseases such as diabetes mellitus, atherosclerosis, and heart failure and elderly people. Locoregional flaps can be an excellent alternative in such cases [4,8,24].

Submental Flap

This flap is a versatile flap. It is an axial pattern flap, the perfusion of which comes from the submental vessels, which branch from the facial artery and vein [4,5]. Some of its advantages are that it is a thin and easy-to-harvest flap, it can be used to cover both defects of the oral cavity (floor of mouth, tongue, and buccal mucosa) and the face (cheek, perioral region), and it can be harvested along with a neck dissection [4,5]. Compared to a free flap, it has common features to RFFF; however, much less surgical time is needed in order to harvest the flap [5]. Care is needed in cases where the submental flap is used for reconstruction in patients with oral cancer and especially in positive neck. However, recent studies have shown that this flap does not compromise the final outcomes of the oncologic patient [5].

Supraclavicular Flap

When it comes to more complex head and neck defects, another important and easy-to-harvest flap is the supraclavicular flap. The anatomy of this fasciocutaneous flap is straightforward. It covers a wide surface from the supraclavicular area to the deltoid region [2,25]. The supraclavicular artery, a branch of the transverse cervical artery, is the main artery that supplies the muscle with blood and can be located in a triangle formed by the clavicle and especially the median portion of it anteriorly, the posterior border of the sternocleidomastoid muscle medially, and the external jugular vein posteriorly [25]. It has a mean diameter of 1.1-1.5 mm and a total length that ranges from 1 to 7 cm [26,27]. A hand-held Doppler is advisable in order to locate the vessel exactly [28]. The transverse cervical vein is mostly responsible for venous drainage [26]. The flap can also be innervated from a nerve deriving from the cervical plexus [25,27]. The minimum dimensions of the flap can be 10-12 by 6-10 cm, ensuring ease of primary closure, whereas the maximum length can be up to 25-30 cm [2]. When raising the flap, it is important to mention that this should be done in a subfascial plane and with respect to the supraclavicular artery in order to maintain the blood supply to the SCIF [28]. Finally, the flap is tunneled under the skin flap at the superior end of it [28]. The donor site is closed in first intention when possible; otherwise, a small skin graft could also be used, if the maximum length of the flap has been harvested [28]. Speaking of the advantages of this flap, it is an easy-to-use and easy-to-harvest flap, pliable enough and with a robust and reliable pedicle, the location of which is predictable using safe anatomical landmarks [25,26]. The one-stage innervated reconstruction and the minimal donor site morbidity along with the several uses of the SCIF make the supraclavicular flap an ideal flap for covering a wide range of head and neck defects (floor of mouth, buccal mucosa, tongue, and soft palate) and a great alternative over free flap reconstruction [26,27,29]. It is also a great alternative to free flaps, especially in patients with comorbidities that would totally benefit from shorter and simpler procedures [27].

The flap is known from the early 90s, when it was used after facial and cervical burn injuries. However, nowadays, the SCIF can be used on several occasions regarding reconstruction after major head and neck cancer ablation, such as parotid carcinoma with skin infiltration or skin cancer of the parotid region and large defects of the oral cavity, and also to restore cervical skin loss especially on recurrent neck disease with exposed carotid vessels and cancer of the oropharynx, hypopharynx, and temporal bone region [2,26,27].

According to the literature, some of the disadvantages of this flap that have been reported are wound dehiscence, mostly at the edge of the flap, necrosis of the distal tip, partial flap necrosis with the need for wound care and debridement, complete flap necrosis (less often), prolonged wound healing in some of the cases, but no functional donor site morbidity, and finally formation of post-operative fistula [2,30-32].

FAMM Flap

When it comes to oral defects, small to medium in volume, the FAMM flap is an excellent alternative. Anatomically, it is an axial flap using the facial artery and can be inferiorly or superiorly based. Vein supply, on the other hand, is mostly random and is based on the venous network traveling at the submucosal plane of the flap [5,33]. Mapping of the facial artery with a handheld Doppler intraorally is of utmost importance in order not to risk the vessel. Calculating its margins, the flap must have a distance of at least 1 cm from the oral commissure, and its base should also be at least 2 cm in width, for unhindered venous drainage and also not to harm the arterial vessel [5,34]. With dimensions of approximately 2 to 3 cm in width and a length covering the distance between the oral commissure and the pterygomandibular raphe, the FAMM flap is an excellent flap for reconstructing a wide spectrum of oral surgical defects and especially difficult palatal fistula and even restoring velopharyngeal insufficiency [5,35,36]. Additionally, it can be used in cleft palate repair [36-38]. A superiorly based flap in specific can be used to cover defects of the anterior palate and the alveolus of the upper jaw, whereas an inferiorly based flap is more ideal for defects of the posterior palate and the floor of the mouth [37,38]. Discussing its advantages, it is a robust axial flap with multiple uses intraorally whether it is used in a superiorly or an inferiorly based pattern. Primary closure of the donor site can be easily implemented in flap sizes up to 3 cm; otherwise, a buccal fat pad can be utilized [39]. It has low morbidity and exceptional cosmetic results [35]. In its disadvantages, we can consider that two-stage reconstruction is mostly needed to release the flap as well as its limited size, making it useful only for oral defects [5].

Comparative Analysis

Indications and Patient Selection

Criteria for free flaps: Free flaps are the gold standard for complex head and neck reconstructions due to their versatility in transferring various tissue types including skin, muscle, and bone from distant donor sites. They are most commonly indicated in extensive oncologic resections requiring three-dimensional (3D) reconstructions, such as mandibular continuity restoration or large composite defects involving the oral cavity and oropharynx. Patient selection relies on several factors such as medical fitness, presence of comorbidities, previous radiotherapy, and surgical complexity. Free flaps are usually reserved for younger, healthier patients due to their prolonged operative time, anesthetic demands, and intensive postoperative monitoring. A recent systematic review emphasized that although free flaps achieve superior reconstructive outcomes, they are associated with longer operating times, higher costs, and increased ICU stays compared to certain locoregional pedicled alternatives [1].

Furthermore, in specialized cancer centers with experienced microsurgical teams, free flap procedures have demonstrated reduced ICU stays and shorter operative times without compromising safety or outcomes, even among older patients [40]. Notably, several studies have reported that age alone should not be a deterrent for free flap surgery, as factors such as frailty, nutritional status, and comorbidity index play a more crucial role in predicting outcomes [41].

Criteria for locoregional flaps: Locoregional flaps are commonly preferred for patients with significant comorbidities, limited physiological reserve, or financially constrained healthcare systems where microvascular reconstruction is not feasible. These flaps, such as the SMIF, supraclavicular artery island flap (SCAIF), and FAMM flap, are pedicled and based on nearby anatomical vascular angiosomes. They are particularly suitable for small to moderate intraoral or facial defects and in salvage scenarios where prior surgeries or radiation therapy preclude the use of free flaps. Studies have consistently shown favorable results with locoregional flaps in elderly and high-risk populations, offering shorter operative times, reduced hospital stays, and lower resource use [1,4].

In low- and middle-income countries (LMICs), locoregional flaps serve as realistic and easily accessible solutions due to limited access to microvascular expertise and infrastructure. A meta-analysis by Hu et al. [8] reported that despite the variability in infrastructure, locoregional reconstructions achieved acceptable oncologic and functional outcomes. Moreover, they facilitate surgical independence in global health missions and enable sustainability through local surgeon training [41].

Emerging protocols integrating flap choice with enhanced recovery after surgery (ERAS) pathways are showing promise [42]. These pathways combine minimally invasive techniques, pain control strategies, and early mobilization to accelerate recovery. In this context, the use of locoregional flaps aligns well with ERAS objectives, particularly for patients who are not suitable for prolonged anesthesia [43].

Surgical Outcomes

The comparison of surgical outcomes between free and locoregional flaps is a crucial factor influencing flap choice. Outcome metrics such as flap survival, return-to-theater rates, need for revision surgery, and donor site morbidity offer valuable insight into the reliability and safety of each reconstructive approach. This section explores how these two techniques perform in real-world clinical settings, especially across varying levels of surgical expertise and institutional support.

Success Rates

Free flaps generally report high success rates, with numerous series citing survival rates exceeding 95%. A series of 127 cases by Başaran et al. demonstrated a flap success rate of 97.6%, reinforcing the efficacy of free flaps when executed by experienced teams [12]. Similarly, Formeister et al. using a machine learning approach confirmed that institutional experience and ischemia time are critical predictors of flap success [44]. The robust outcomes are also reflected in complex reconstructions, including those involving double flaps or osseous tissue. In elderly patients, studies such as those by Sukato et al. have further validated high success rates, showing no significant increase in surgical failure compared to younger counterparts [45].

Locoregional flaps also show robust survival outcomes when appropriately selected. In a meta-analysis comparing SMIF to free flaps, Jørgensen et al. found no significant differences in total flap loss or recurrence rates, with SMIF offering the advantage of shorter hospitalization and operative times [4]. Gabrysz-Forget et al. likewise concluded that SMIF and SCAIF offered comparable success to free flaps in carefully selected patients [1]. Their utility has been further enhanced by advances in anatomical understanding and refinements in surgical technique.

Complication Rates

While both techniques are generally safe, their complication profiles differ. Free flaps, due to longer operative times and microvascular anastomoses, carry risks of thrombosis, ischemia, and the need for take-back operations. Formeister et al. identified ischemia time and patient age as significant predictors of complications, with a take-back rate of 16.2% in their cohort [44]. Other studies, such as Patel et al., have shown that venous complications are more common than arterial ones, and early re-exploration within the first 24 hours significantly improves salvage rates [5]. Salvage success with re-exploration or second free flaps remains high-over 90%-indicating that aggressive management of complications can yield good outcomes [45].

Locoregional flaps, though avoiding microsurgical risks, can suffer from issues such as partial flap necrosis, especially in patients with a history of neck dissection or radiation. Jørgensen et al. reported a higher rate of partial necrosis in SMIF (5%) compared to free flaps, although complete flap loss remained rare [4]. Hematoma and wound dehiscence are common but usually manageable, especially with improved perioperative care protocols. Additionally, anticoagulant and antiaggregant use has been linked to higher hematoma rates in locoregional reconstructions [12].

Functional and Aesthetic Results

The evaluation of functional and aesthetic outcomes is fundamental to determining long-term success in head and neck reconstruction. Effective speech, swallowing, facial movement, and cosmetic appearance significantly impact patients’ post-treatment quality of life. These considerations often influence the choice of flap just as much as surgical feasibility, particularly in cases involving the oral cavity, oropharynx, or facial structures.

Functional recovery: speech, swallowing, and other functional outcomes: Functional outcomes, particularly speech and swallowing, are critical in head and neck reconstruction. Free flaps such as the RFFF and ALT are pliable and well-suited for intraoral defects, promoting excellent speech and deglutition recovery. Başaran et al. highlighted the suitability of RFFF for subtotal glossectomy with favorable functional results [12].

FAMM flaps also achieve good functional recovery for smaller oral cavity defects. Ibrahim et al. showed no significant differences in swallowing or speech outcomes between FAMM and RFFF groups, despite the FAMM group benefiting from shorter operations and reduced morbidity [46]. Thus, when used in suitable indications, locoregional flaps do not compromise functional rehabilitation. Additionally, a review by Sandilands et al. noted that FAMM flaps offer superior preservation of mucosal sensitivity and neuromuscular function, further enhancing functional outcomes [47].

Aesthetic outcomes: cosmetic results and patient satisfaction: Aesthetic considerations are important for patient quality of life. Free flaps, especially osseous types like fibula or scapula flaps, allow for 3D reconstruction and superior contouring, particularly in mandibular defects. However, donor site morbidity, such as forearm or leg scarring, must be acknowledged.

Locoregional flaps, especially SMIF and SCAIF, provide better color and texture match with minimal donor site disfigurement. Patel et al. noted that SCAIF provides excellent aesthetic outcomes in the cervical and facial region without the bulk of PMMF flaps [5]. Aesthetic satisfaction is often higher with these options in carefully chosen anterior facial defects. Moreover, in elderly patients where quality of life and rapid recovery are prioritized over extensive reconstruction, locoregional flaps offer balanced aesthetic and functional outcomes with minimal invasiveness [48].

Cost and Resource Utilization

Economic constraints and healthcare resource availability are crucial when choosing reconstructive strategies. Free and locoregional flaps differ significantly in their requirements for specialized equipment, personnel, and postoperative monitoring. These differences influence their accessibility across healthcare settings from high-volume tertiary centers to regional hospitals and LMICs. A comprehensive analysis of cost and resource utilization facilitates decision-making that upholds clinical efficacy while ensuring economic sustainability.

Economic considerations: The cost difference between free and locoregional flaps is substantial. Free flaps require long operative times, microvascular instruments, ICU monitoring, and multidisciplinary teams, all of which escalate costs. Standalone cancer centers could reduce ICU time by over one day, saving approximately $223,000 in total; nevertheless, free flap surgery remained resource-intensive [40].

Gabrysz-Forget et al. observed that SMIF and SCAIF reduced both surgical and hospitalization costs without sacrificing outcomes, making them economically favorable alternatives in appropriate contexts [1]. Similarly, Ibrahim et al. demonstrated a per-case cost reduction of over 40% when choosing FAMM over RFFF [46]. In systems with constrained health budgets, these savings can translate into broader access to reconstructive care without compromising quality [48].

Resource requirements: Free flap surgery necessitates specialized training, operative microscopes, and access to perioperative critical care, limiting its availability in LMICs and smaller institutions. In contrast, locoregional flaps can be performed by general head and neck surgeons with basic instrumentation and local anesthesia in some cases.

Hu et al. emphasized the practicality of locoregional flaps in LMICs, noting acceptable complication rates and oncologic control when combined with structured surgical training and preoperative planning [8]. The use of locoregional techniques in outreach and humanitarian missions has further cemented their value as versatile, scalable, and sustainable reconstructive options [49].

Advances in microvascular free tissue transfer

Technological Innovations

Recent advancements in surgical tools and techniques have revolutionized microvascular free tissue transfer for head and neck reconstruction, enhancing precision and outcomes. High-resolution operating microscopes with 3D visualization and robotic-assisted systems enable meticulous anastomosis of vessels as small as 0.8 mm, critical for complex reconstructions like those involving the mandible or pharynx [22,50]. Intraoperative imaging, particularly near-infrared fluorescence angiography with indocyanine green (ICG), has become pivotal in assessing real-time flap perfusion and significantly reducing flap failure rates in high-volume centers [51]. Additionally, 3D-printed patient-specific vascular templates streamline preoperative planning, ensuring accurate flap design for defects involving the oral cavity or skull base. A recent study demonstrated that ICG angiography significantly improved flap survival rates in head and neck reconstructions, while highlighting the role of 3D-printed guides in reducing operative time and improving aesthetic outcomes in the literature [52,53].

Training and Expertise

The development of surgical training programs has significantly elevated expertise in microvascular surgery for head and neck reconstruction, ensuring proficiency in managing complex defects. Simulation-based training, including virtual reality platforms and high-fidelity anastomosis models, allows trainees to master techniques like supermicrosurgery before operating on patients [54]. Specialized microsurgery fellowships, often spanning 1-2 years, provide hands-on experience with flaps such as the ALT and fibula free flap, commonly used in head and neck cases. Global collaboration facilitates skill-sharing, particularly for challenging reconstructions like osteocutaneous flaps. A 2017 review by Lo Nigro et al. underscored the importance of interdisciplinary training in improving functional outcomes for head and neck cancer patients, while a 2024 study by Shahrezaei et al. emphasized the efficacy of simulation-based training in reducing intraoperative errors among fellows [55,56].

Future directions

Looking ahead, the future of microvascular free tissue transfer in head and neck reconstruction lies in integrating cutting-edge technologies and personalized approaches to optimize outcomes. Tissue engineering, combined with vascularized bioengineered scaffolds, holds promise for creating custom flaps that minimize donor site morbidity, particularly for soft tissue defects [57]. Artificial intelligence (AI) is emerging as a tool for predicting flap viability and guiding intraoperative decisions, potentially reducing complications like partial flap necrosis [44]. Additionally, regenerative medicine, including stem cell therapies, could enhance tissue integration in irradiated fields, a common challenge in head and neck cases. Preclinical data suggest that stem cell-enhanced flaps can improve wound healing [58]. These innovations, alongside efforts to expand access in low-resource settings, will shape the next era of head and neck reconstruction.

Discussion

Interpretation of Findings

The comparative analysis of free tissue transfer and locoregional flaps for reconstructing small to moderate head and neck defects reveals distinct advantages and trade-offs for each approach. Microvascular free flaps, such as the RFFF and ALT, demonstrate superior versatility, enabling precise 3D reconstruction and excellent functional outcomes, particularly for speech and swallowing. Their high success rates, often exceeding 95%, underscore their reliability in complex cases, especially when supported by experienced microsurgical teams [59]. In contrast, locoregional flaps, including the SMIF, SCAIF, and FAMM flap, offer comparable success rates for smaller defects, with the added benefits of shorter operative times and reduced resource demands. However, locoregional and free tissue flaps may face higher rates of complicated healing, particularly in patients with comorbidities [60]. Considering all that, the final choice between these techniques hinges on defect complexity, patient comorbidities, and institutional capabilities, with free flaps excelling in extensive resections and locoregional flaps serving as effective alternatives for high-risk patients or resource-limited settings.

Clinical Implications

The findings have significant implications for clinical decision-making in head and neck reconstruction. Free flaps should be prioritized in cases requiring extensive tissue replacement or bone reconstruction, such as mandibular defects, due to their adaptability and robust functional outcomes. However, for patients with significant comorbidities, advanced age, or limited physiological reserve, locoregional flaps provide a safer and more practical option, reducing operative time and postoperative complications [61]. In LMICs, where access to microvascular expertise and infrastructure may be limited, locoregional flaps offer a sustainable and cost-effective solution, enabling surgeons to achieve acceptable oncologic and functional outcomes. On the other hand, the integration of ERAS protocols further supports the use of locoregional flaps in high-risk populations, promoting faster recovery and reduced hospital stays [62]. Clinicians must weigh these factors alongside patient-specific considerations, such as prior radiation or nutritional status, to optimize reconstructive strategies.

Limitations of the review

This review has several limitations that warrant consideration. First, reliance on existing literature introduces variability in study designs, patient populations, and outcome metrics, which may affect the generalizability of findings. Many studies included in the analysis were retrospective, potentially introducing selection bias and limiting the ability to control for confounding factors such as surgeon experience or institutional protocols. Additionally, the review primarily focuses on small to moderate defects, potentially underrepresenting the nuances of larger or composite defects where free flaps may have a more pronounced advantage. Data from LMICs are also limited, which may skew the economic and resource utilization analysis toward high-resource settings. Finally, the review does not extensively address long-term functional outcomes, such as quality-of-life metrics beyond speech and swallowing, which are critical for comprehensive patient-centered care.

Recommendations for practice

Based on the evidence, clinicians should adopt a tailored approach to head and neck reconstruction, guided by defect characteristics, patient health status, and institutional resources. For complex defects requiring bone or large soft tissue reconstruction, microvascular free flaps, such as RFFF or fibula flaps, should be the first choice in medically fit patients, supported by intraoperative tools like ICG angiography to enhance flap survival. In contrast, locoregional flaps, such as SMIF or FAMM, are recommended for small to moderate intraoral defects, particularly in elderly or comorbid patients, to minimize operative risks and recovery time. Surgeons in resource-constrained settings should prioritize training in locoregional flap techniques to ensure sustainable reconstructive options. Additionally, incorporating ERAS protocols and preoperative frailty assessments can further optimize outcomes across both flap types. Multidisciplinary collaboration and ongoing microsurgical training are essential to balance efficacy, safety, and accessibility in head and neck reconstruction.

## Conclusions

This narrative review highlights the distinct roles of microvascular free flaps and locoregional flaps in reconstructing small to moderate defects in the head and neck region. Free flaps, such as the RFFF and ALT, are the gold standard for complex reconstructions, offering versatility, high success rates, and slightly superior functional outcomes, particularly for speech, swallowing, and 3D tissue replacement. However, their resource-intensive nature, prolonged operative times, and need for specialized expertise limit their accessibility, especially in LMICs. Locoregional flaps, including the SMIF, SCAIF, and FAMM flap, provide effective alternatives for smaller defects, with comparable success rates, shorter operative times, and lower costs. These flaps are particularly suitable for high-risk patients with comorbidities or in resource-constrained settings. The choice between flap types should be guided by defect complexity, patient health, and institutional capabilities, with both approaches achieving favorable functional and aesthetic outcomes when appropriately selected.

The future of head and neck reconstruction lies in advancing technological innovations, refining surgical training, and addressing disparities in access to care. Emerging technologies, such as tissue-engineered flaps, AI for intraoperative decision-making, and stem cell therapies, hold promise for minimizing donor site morbidity and improving outcomes in challenging cases. Ongoing research is needed to optimize these innovations, particularly in integrating bioengineered scaffolds with vascularized tissue for personalized reconstruction.
